# Natural history in hereditary spastic paraplegias: real-world data from an Austrian cohort

**DOI:** 10.1007/s00415-025-13606-y

**Published:** 2026-01-26

**Authors:** Matthias Amprosi, Elisabetta Indelicato, Andreas Eigentler, Daniel Boesch, Josef Fritz, Wolfgang Nachbauer, Sylvia Boesch

**Affiliations:** 1 Center for Rare Movement Disorders, Department of Neurology, Medical University of Innsbruck, Innsbruck, Austria; 2 Institute of Clinical Epidemiology, Public Health, Health Economics, Medical Statistics and Informatics, Medical University of Innsbruck, Innsbruck, Austria

**Keywords:** Hereditary spastic paraplegias, Natural history, Outcome measurements, Spastic Paraplegia Rating Scale (SPRS), Trial readiness

## Abstract

**Introduction:**

Hereditary spastic paraplegias (HSP) are rare inherited neurodegenerative disorders characterized by progressive lower limb spasticity and weakness. This study aimed to characterize an Austrian HSP cohort and prospectively assess disease progression using the Spastic Paraplegia Rating Scale (SPRS), addressing the knowledge gap regarding its longitudinal capabilities in a real-world setting.

**Methods:**

Data from 126 patients were collected at the Center for Rare Movement Disorders Innsbruck. Baseline clinical data were available for 103 individuals. Follow-up extended up to 5 years (mean 2.3 ± 1.9). Disease severity was assessed with the SPRS, and longitudinal progression analyzed using generalized linear mixed models.

**Results:**

The cohort (64.3% male, mean age 47.1 years) included 54.8% patients with complicated HSP. Genetic confirmation was achieved in 54.0%, with *SPAST* being the most frequent genotype (36.8%). Mean baseline SPRS was 18.2 points. SPRS scores increased significantly with disease duration, with an overall annual progression of 0.9 points (p < 0.001). Progression was faster in complicated versus pure HSP (1.3 vs. 0.6 points/year; p < 0.001). Most patients received symptomatic medication (69.8%) and neurorehabilitation (84.1%).

**Conclusion:**

This study provides comprehensive real-world data on HSP from an Austrian cohort, including clinical, genetic, management, and imaging findings. We present the first prospective assessment of SPRS progression in a natural history cohort, revealing significant longitudinal change. Taken together, our findings may contribute to the design of future therapeutic trials in HSP.

**Supplementary Information:**

The online version contains supplementary material available at 10.1007/s00415-025-13606-y.

## Introduction

Hereditary spastic paraplegias (HSP) are a group of rare inherited neurodegenerative disorders characterized by progressive spasticity and weakness of the lower limbs. The global prevalence has been estimated to be 3.6 per 100,000 individuals [1].

To date, more than 80 different genes associated with HSP have been identified [2]. Variants in these genes disrupt various cellular processes, including axonal transport, mitochondrial function, and lipid metabolism, ultimately leading to degeneration of the corticospinal tract [2]. The inheritance pattern of HSP encompass autosomal dominant (AD), autosomal recessive (AR), X-linked, and mitochondrial modes of transmission [3, 4].

Clinically, HSP can be classified into pure (pHSP) and complicated (cHSP) forms. Pure HSP is characterized by the core features of lower limb spasticity, weakness, bladder disturbances, and decreased vibration sense. Complicated HSP presents with additional clinical features such as cognitive impairment, epilepsy, or ataxia [5]. The Spastic Paraplegia Rating Scale (SPRS) is a validated rating scale to depict disease severity in HSPs [6].

In this prospective study, we (1) recruited and characterize a large Austrian HSP cohort of more than 126 patients (102 unrelated families) with HSP, including detailed clinical, genetic, and real-world data on the management and care from the National Reference Center for Rare Movement Disorders Innsbruck and (2) assessed follow-up (FU) data in all the above-mentioned aspects, to define the progression of disease in HSPs.

## Methods

The present study was conducted at the “Center for Rare Movement Disorders Innsbruck” (CRMDI). The CRMDI is a national reference center as well as a member of the European Reference Network for Rare Neurological Diseases (Project ID No. 739510) and receives referrals from all over Austria and South Tyrol (Italy). All subjects with a clinical diagnosis of HSP followed between January 2000 and January 2025 at our center were included in the analysis.

### Patients

In total, 126 patients from 102 families were included, including 88 (69.8%) unrelated individuals.

Baseline visits with detailed clinical data, defined by the presence of a Spastic Paraplegia Rating Scale (SPRS) score, were available for 103 patients. Annual follow-up visits (FU) were planned for all patients. The longest FU time in this ongoing cohort study was 5 years and available in 14 patients. Genetic testing was performed on a routine clinical basis in certified laboratories. Initially, a sequential genetic testing approach was applied. In uncomplicated cases, single-gene analyses were performed first and, if negative, followed by additional testing (primarily NGS gene panels; WES and MLPA in selected cases) when indicated. Since the publication of the diagnostic pathway for HSP by the ERN-RND in 2019, we have followed these recommendations [7]. We now perform single-gene analyses only in specific cases, such as in uncomplicated forms with a suspected SPG4 variant or for confirmation of a known familial mutation. All other patients are now examined using next generation sequencing (NGS)-based gene panels or whole-exome sequencing (WES).

The SPRS was used to assess disease severity. This score ranges from 0 (no signs or symptoms) to 52 points (maximum disease severity) and consists of 13 items. Every item (e.g., gait, spasticity, weakness) has zero to four points [6]. Furthermore the 4-Stage Scale of Motor Disability (4SMD) was administered. This scale was first described in 2009 and ranges from 0 to 4: 0—no signs and symptoms, 1—mild symptoms and signs at examination, ambulatory without an aid, 2—walking without an aid, unable to run, 3—walking with an aid, and 4—wheelchair bound [8].

We assessed participants’ educational attainment using the International Standard Classification of Education (ISCED) of the United Nations Educational, Scientific and Cultural Organization. For analysis, we adapted the scale by summarizing ISCED levels 5 to 8 as "academics" to reflect higher education attainment. ISCED0 corresponds to early childhood education, ISCED1 to primary education, ISCED2 to lower secondary, ISCED3 to upper secondary, ISCED4 to post-secondary non-tertiary, and ISCED5–8 encompass short-cycle tertiary, bachelor’s, master’s, and doctoral or equivalent levels, respectively [9].

### Statistical analysis

Statistical analyses were performed using SPSS software (IBM Corp. Released 2023. IBM SPSS Statistics for Windows, Version 29.0.2.0 Armonk, NY: IBM Corp). The threshold for statistical significance was set at *p* < 0.05 (two-sided). Data were reported as mean and standard deviation (SD), median and interquartile range (IQR), or frequencies and percentages, as appropriate. The Mann–Whitney U test and the chi-squared test were applied to assess subgroup comparisons, and Spearman's rank correlation coefficient was used to address correlations.

We conducted a generalized linear mixed model (GLMM) analysis to examine changes in SPRS scores over time. In the first step, we analysed the overall changes in SPRS scores across all visits for all participants using only the timepoint of the visit as a fixed effect. We additionally repeated the model including only participants who completed the FU4 visit (complete-case-approach) to exclude potential bias due to missed visits. In the second step, we included the effect of the HSP form (pHSP vs. cHSP) and its interaction with the timepoint (timepoint × form) to assess group differences in the longitudinal trajectories. Both models included a random intercept for participants to account for within-subject dependencies. In a third step, we added the effects of age at onset and disease duration to examine their influence on SPRS progression. Finally, we repeated the general model including only SPG4 patients, which was the only subcohort allowing a meaningful subgroup analysis. Because the SPRS score was positively skewed and not normally distributed, the model was calculated with gamma family and log-link function as well as likelihood ratio tests. The Last Observation Carried Forward method was applied to handle missing SPRS scores. In this approach, the last available score of a participant is used to replace a missing value at a subsequent time point. A maximum of two missing values per participant were imputed using this approach.

### Ethics

This study was conducted in accordance with the principles of the Declaration of Helsinki. This study was approved by the institutional review board (Vote Nr: 1255/2018). All subjects included in the prospective study provided written informed consent prior to their inclusion.

## Results

### Demographics

A total of 126 patients, 81 (64.3%) of whom were male, were included in the study. Of these patients, 38 (30.2%) were related, resulting in a total of 102 unrelated families. Four participants (3.2%) were younger than 18 years at the time of the baseline visit. Seventy (55.6%) of the subjects had a positive family history and where thus classified as familial and 56 (44.4%) as isolated cases. Complicated HSP (cHSP) was diagnosed in 69 subjects (54.8%). The mean age of the total cohort was 47.1 years (SD ± 15.7) at the first assessment, with pure HSP patients (pHSP; 52.7 ± 13.7) being significantly older than patients with complicated HSP (cHSP; 42.4 ± 15.8; *p* < 0.001). The age distribution between males and females was similar (male 47.4 ± 14.0 versus female 46.4 ± 18; p = 0.978). The average age at onset for the entire cohort was 29.9 ± 17.5 years, with pHSP patients showing a significantly later onset than cHSP patients (35.2 ± 16.5 vs. 25.4 ± 17.1; *p* = 0.002). The age at onset was not significantly different between the sexes (*p* = 0.104).

Average disease duration of the total cohort was 17.2 years (SD ± 12.4) and while there was no significant difference between the disease forms (p = 0.928), males had a longer disease duration compared to females (19.4 ± 13.2 vs.13.2 ± 10.0; p = 0.008). Childhood development was unremarkable in 99 subjects (78.6%). Patients with cHSP showed a higher rate of developmental delay than those with pHSP (n = 21 [30.4%] versus 6 [10.5%] p = 0.011). There was no difference between the sexes concerning the frequency of developmental delay (p = 0.099).

Further demographic details are shown in Table 1.

**Table 1:** Demographics and clinical characteristics

	Total	pHSP	cHSP	p-value	Male	Female	p-value
n (%)	126 (100)	57 (45.2)	69 (54.8)		81 (64.3)	45 (35.7)	
Age, mean (SD)	47.1 (15.7)	52.7 (13.7)	42.4 (15.8)	**< 0.001**	47.4 (14)	46.4 (18.5)	0.974
Age at onset, mean (SD)	29.9 (17.5)	35.2 (16.5)	25.4 (17.1)	**0.002**	28 (17.5)	33.2 (17.2)	0.108
Disease duration, mean (SD)	17.2 (12.4)	17.4 (13.4)	17 (11.7)	0.912	19.4 (13.2)	13.2 (10)	**0.008**
Consanguinity, n (%)							
No	119 (94.4)	57 (100)	62 (89.9)	**0.014**	76 (93.8)	43 (95.6)	0.685
Yes	7 (5.6)	0 (0)	7 (10.1)		5 (6.2)	2 (4.4)	
Genetically confirmed, n (%)							
No	53 (42.1)	18 (31.6)	35 (50.7)	0.079	37 (45.7)	16 (35.6)	0.345
Yes	68 (54)	37 (64.9)	31 (44.9)		40 (49.4)	28 (62.2)	
VUS	5 (4)	2 (3.5)	3 (4.3)		4 (4.9)	1 (2.2)	
Mode of inheritance, n (%)							
Autosomal dominant	41 (32.5)	28 (49.1)	13 (18.8)	**< 0.001**	27 (33.3)	14 (31.1)	0.859
Autosomal recessive	37 (29.4)	7 (12.3)	30 (43.5)		23 (28.4)	14 (31.1)	
Sporadic	42 (33.3)	19 (33.3)	23 (33.3)		26 (32.1)	16 (35.6)	
X-linked	5 (4)	3 (5.3)	2 (2.9)		4 (4.9)	1 (2.2)	
Mitochondrial	1 (0.8)	0 (0)	1 (1.4)		1 (1.2)	0 (0)	
Childhood development, n (%)							
Unremarkable	99 (78.6)	51 (89.5)	48 (69.6)	**0.011**	60 (74.1)	39 (86.7)	0.131
Abnormal	27 (21.4)	6 (10.5)	21 (30.4)		21 (25.9)	6 (13.3)	
4SMD, n (%)							
Mild symptoms and signs, ambulatory without aid	15 (11.9)	9 (15.8)	6 (8.7)	**0.001**	10 (12.3)	5 (11.1)	0.663
Walking without an aid, unable to run	53 (42.1)	30 (52.6)	23 (33.3)		31 (38.3)	22 (48.9)	
Walking with an aid	36 (28.6)	16 (28.1)	20 (29)		24 (29.6)	12 (26.7)	
Wheelchair bound	22 (17.5)	2 (3.5)	20 (29)		16 (19.8)	6 (13.3)	
Scales and scores, mean (SD)							
SPRS score	18.2 (10.5)	15.4 (8)	20.2 (11.6)	**0.071**	18.6 (10.9)	17.4 (9.8)	0.703

Demographics and clinical characteristics of the total cohort, stratified by HSP form (pHSP vs. cHSP) and sex (male vs. female)

Values are presented as numbers (%) or mean (SD), as indicated. p-values are shown in separate columns; significant differences between HSP forms and sexes are indicated in bold (p < 0.05). Mode of inheritance was assessed using an integrated assessment of pedigree information and genetic testing results: Confirmed pathogenic variants following AD, AR, X-linked, or mitochondrial transmission were classified accordingly, independent of familial occurrence. In cases without an identified genetic variant, classification was based on pedigree information and the presumed inheritance pattern, assigning patients as AD, AR or sporadic as appropriate

Significant differences between HSP forms and sexes are indicated in bold (*p* < 0.05)

*pHSP* pure hereditary spastic paraplegia, *cHSP* complicated hereditary spastic paraplegia, *VUS* variant of uncertain significance, *SPRS* spastic paraplegia rating scale

Genetic confirmation was achieved in 68 (54.0%) of the patients and 50 (49.0%) of the families, with no significant differences in the diagnostic rates across sexes (*p* = 0.345) or pHSP versus cHSP (*p* = 0.079). Among familial HSP cases, 34 of 46 index patients (73.9%) had a genetically confirmed diagnosis, compared with 15 of 56 index patients (26.8%) in the sporadic group. AD, AR, and sporadic inheritance accounted for approximately one-third of the cases, whereas x-chromosomal and mitochondrial inheritance were only seen in a small number of subjects (n = 5 and 1 respectively). There was a significant difference in the modes of inheritance between pHSP and cHSP; cHSP showed higher AR inheritance (n = 30 [43.5%] vs. n = 7 [12.3%]; p < 0.001), whereas pHSP, dominated by *SPG4/SPAST*, followed AD inheritance (n = 28 [59.1%] vs. n = 13 [18.8%] p < 0.001). Consanguinity was observed in seven (5.6%) subjects, exclusively in patients with cHSP (n = 7 [10.1%]). In genetically assigned cases, variants in SPAST gene (spastic paraplegia gene [SPG4]) were the predominant genotype, with 25 (36.8%) cases. *SPG11* was the second most common gene affected in 10 (14.7%) patients, followed by *SPG7* in six (8.8%) subjects. Variants of uncertain significance (VUS) were found in 5 patients (4.0%).

**Fig. 1:**
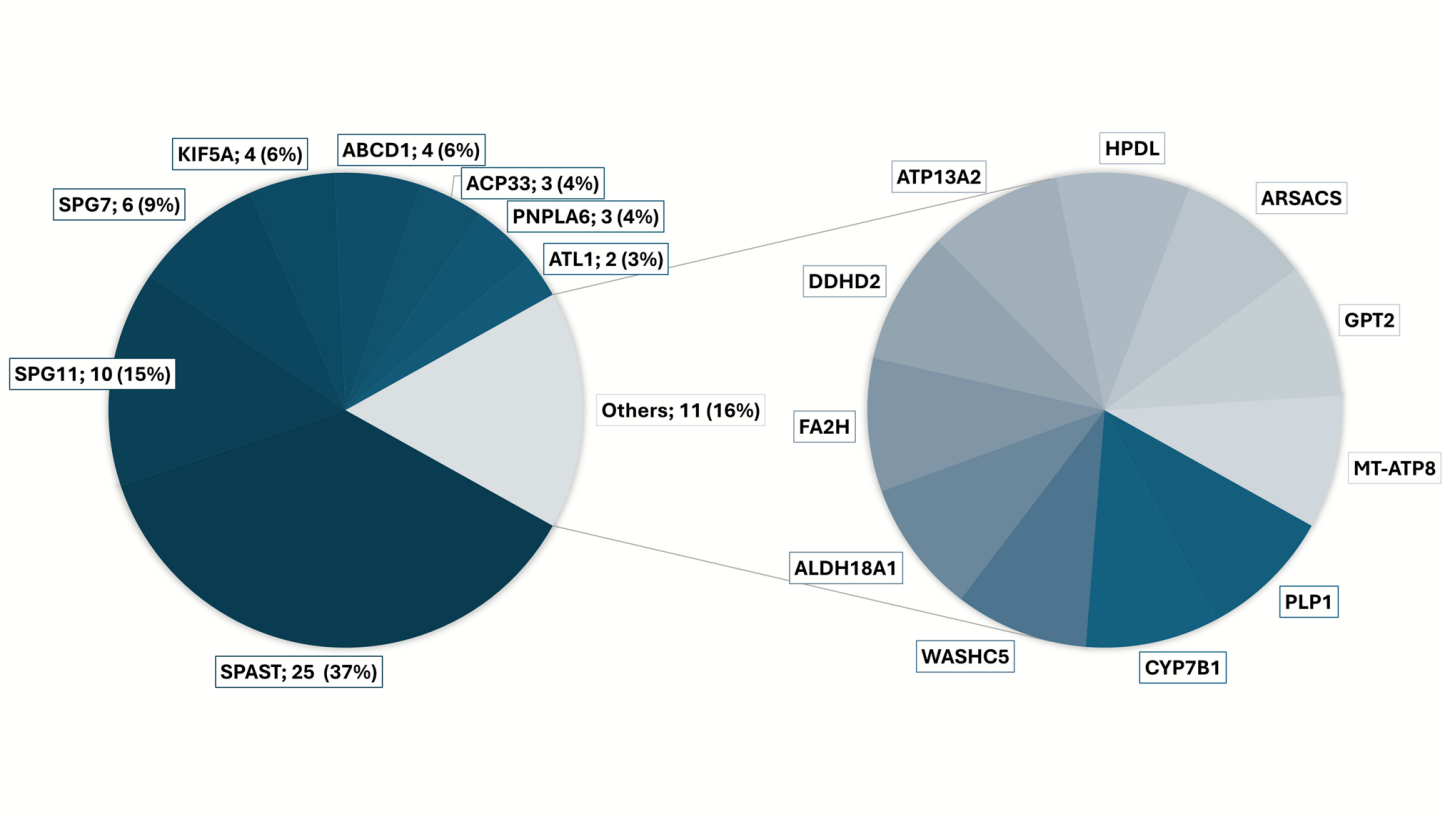
**Genetic composition of the cohort.** Genetic composition of the cohort, displayed as a pie chart. The chart on the left shows all genes identified more than once, with gene names followed by the number of individuals affected (percentage). The chart on the right displays genes that were each found only once in the cohort. For a full list of variants see Table 2. *SPG* spastic paraplegia gene

For a detailed overview of the prevalence of the different genotypes, see Fig. 1 and Table 2.

**Table 2:** Overview of all genetically confirmed families included in the study

Family	n per family	Sex	Inherit -ance	AOA	Form	Gene	Sequence	Variant allele 1	Variant allele 2	Known or novel	Assessment of pathogenicity
1	1	F	AR	11	cHSP	GPT2	NM_133443.4	c.1319A > G, p.(Tyr440Cys)	c.1319A > G, p.(Tyr440Cys)	Novel	Likely pathogenic
2	1	F	X-L	40	pHSP	ABCD1	NM_000033.4	c.1679C > T, p.(Pro560Leu)		Known	Pathogenic
3	2	M	AR	5	cHSP	SPG11	NM_025137.4	c.1621C > T, p.(Gln541*); c.6526 T > C, p.(Phe2176Leu)	c.1621C > T, p.(Gln541*); c.6526 T > C, p.(Phe2176Leu)	Known/known	Pathogenic/pathogenic
4	1	F	AD	20	pHSP	SPAST	NM_014946.4	c.1120C > G, p.(Pro734Ala)		Novel	Likely pathogenic
5	1	M	AR	32	pHSP	CYP7B1	NM_004820.5	c.1328G > C, p.(Gly443Ala)	c.425A > G, p.(Tyr142Cys)	Known/known	Pathogenic/likely pathogenic
6	1	M	AR	6	cHSP	SPG11	NM_025137.4	c.4790G > A, p.Trp1597*)	c.4790G > A, p.(Trp1597*)	Known/known	Pathogenic/pathogenic
7	2	M	X-L	20	pHSP	ABCD1	NM_000033.4	c.1165C > G, p.(Arg389Gly)		Known	Pathogenic
8	5	M	AD	30	pHSP	SPAST	NM_014946.4	c.158delT, p.(Phe53Serfs*7)		Known	Pathogenic
9	1	M	AD	64	pHSP	ATL1	NM_015915.5	c.883C > T, (p.Arg239Cys)		Known	Pathogenic
10	1	M	AD	3	pHSP	ATL1	NM_015915.5	c.883C > T, (p.Arg239Cys)		Known	Pathogenic
11	1	M	AR	2	cHSP	SPG11	NM_025137.4	c.4307_4308del, (p.Gln1436Argfs*7)	c.6829G > A, p.(Glu2277Lys)	Known/novel	Pathogenic/likely pathogenic
12	1	M	AR	47	pHSP	B4GALNT1	NM_001478.4	c.943G > A, p.(Gly315Arg)	c.943G > A, p.(Gly315Arg)	Novel/novel	VUS
13	1	F	AR	35	cHSP	ACP33	NM_016630.3	c.487delA, p.(Ile163*)	c.487delA, p.(Ile163*)	Known/known	Pathogenic/pathogenic
14	1	F	AD	47	pHSP	SPAST	NM_014946.4	c.158delT, p.(Phe53Serfs*7)		Known	Pathogenic
15	2	F	AR	6	cHSP	SPG11	NM_025137.3	c.2990 T > A, p.(Leu997Gln)	c.4878delTT, p.(Phe1626fs*2)	Known/novel	Pathogenic/pathogenic
16	1	M	AR	44	cHSP	SPG7	NM_003119.4	c.1529C > T, p.(Ala510Val)	c.2225A > G, p.(Asp742Gly)	Known/novel	Pathogenic/pathogenic
17	2	M	AR	8	cHSP	PNPLA6	NM_001166114.2	c.1853C > T, p(.Ala618Val)	c.2810G > A, p.(Gly937Glu)	Known/known	Pathogenic/pathogenic
18	1	M	AD	2	pHSP	SPAST	NM_014946.4	Deletion exon 8		Known	Pathogenic
19	2	F	AD	22	pHSP	KIF5A	NM_004984.4	c.751G > A, p.(Glu251Lys)		Known	Pathogenic
20	1	F	AR	19	cHSP	HPDL	NM_032756.4	c.569C > T, p.(Pro190Leu)	c.569C > T, p.(Pro190Leu)	Known/known	Pathogenic/pathogenic
21	2	M	AR	10	cHSP	ACP33	NM_016630.3	c.118C > T, p.(Arg40*)	c.153delT, p.(Val52fs)	Known/known	Pathogenic/pathogenic
22	2	M	AR	17	cHSP	SPG11	NM_025137.4	c.2431C > T, p.(Gln811*)	c.4877_4878delTT, p.(Phe1626fs)	Known/known	Pathogenic/pathogenic
23	1	M	AR	2	cHSP	ARSACS	NM_014363.6	Deletion of 1158 kb including SACS	c.5151dupA; p.(Ser1718Ilefs*20)	Known/known	Pathogenic/pathogenic
24	1	M	X-L	16	cHSP	ABCD1	NM_000033.2	c.1817C > T, p.(Ser606Leu)		Known	Pathogenic
25	1	F	AD	7	cHSP	ALDH18A1	NM_002860.4	c.416A > G, p.(Gln139Arg)		Novel	VUS
26	1	M	AR	30	cHSP	DDHD2	NM_015214.2	c.1547G > A, p.(Arg516Gln)	c.1547G > A, p.(Arg516Gln)	Novel/novel	Likely pathogenic/likely pathogenic
27	1	M	AR	11	cHSP	SPG11	NM_025137.4	c.1951C > T, p.(Arg651*)	c.1951C > T, p.(Arg651*)	Known/known	Pathogenic/pathogenic
28	1	M	AD	20	cHSP	KIF5A	NM_004984.4	c.827A > G, p.(Tyr276Cys)		Known	Pathogenic
29	1	M	AR	40	cHSP	AP4S1	NM_001128126.3	c.413delG, p.(Gly138Alafs*6)	c.413delG, p.(Gly138Alafs*6)	Novel/novel	VUS
30	1	M	AR	44	pHSP	FA2H	NM_024306.4	c.910G > T, p.(Gly304Cys)	c.806G > A, p.(Arg269His)	Known/known	Pathogenic/pathogenic
31	1	M	AD	40	pHSP	SPAST	NM_014946.4	c.1242delA, p.(Lys414Asnfs*10)		Novel	Pathogenic
32	1	M	AR	50	pHSP	SPG7	NM_003119.4	c.1045G > A, p.(Gly349Ser)	c.1408C > T, p.(Arg470*)	Known/known	Pathogenic/pathogenic
33	1	F	AR	61	cHSP	SPG7	NM_003119.4	c.233 T > A, p.(Leu78*)	c.1045G > A, p.(gly349Ser)	Known/known	Pathogenic/pathogenic
34	1	M	Mito	20	cHSP	MT-ATP8	n.a	m.8490_8490_TA		Novel	Likely pathogenic
35	5	F	AD	37	pHSP	SPAST	NM_014946.4	c.549dup, p.(Asn184*)		Known	Pathogenic
36	2	F	AD	55	cHSP	SPAST	NM_014946.4	c.1216A > G, p.(Ile406Val)		Known	Pathogenic
37	1	F	AD	50	pHSP	SPAST	NM_014946.4	Deletion exon 1		Known	Pathogenic
38	1	M	X-L	1	cHSP	PLP1	NC_000023.1	g.(103477300_103776506)_ (104039333_ 104,817,980)dup		Known	Pathogenic
39	1	F	AR	53	pHSP	SPG7	NM_003119.4	c.1529C > T, p.(Ala510Val)	c.1529C > T, p.(Ala510Val)	Known/known	Pathogenic/pathogenic
40	1	F	AR	26	pHSP	PNPLA6	NM_006702.4	c.721C > T, p.(Arg241Trp)	c.2944_2947dup, p.(Arg983Glnfs*38)	Novel/known	Likely pathogenic/pathogenic
41	1	M	X-L	50	pHSP	ABCD1	NM_000033.4	c.1901C > T, p.(Ala634Val)		Novel	Pathogenic
42	1	F	AR	18	cHSP	SPG7	NM_003119.4	c.1529C > T, p.(Ala510Val)	c.1749G > C, p.(Trp538Cys)	Known/known	Pathogenic/pathogenic
43	1	F	AR	15	cHSP	SPG11	NM_025137.4	c.2612dupG, p.(Ser871Argfs*11)	c.4434G > T, p.(Trp1478Cys)	Known/known	Likely pathogenic
44	1	M	AD	30	cHSP	KIAA0196	NM_003119.4	c.1891C > A, p.(Pro631Thr)		Novel	Likely pathogenic
45	1	F	AD	57	cHSP	SPAST	NM_014946.4	c.1540A > G, p.(Arg514Gly)		Known	Pathogenic
46	1	M	AD	47	pHSP	SPAST	NM_014946.4	c.1245 + 1G > A,		Known	Pathogenic
47	1	M	AR	40	pHSP	SPG7	NM_003119.4	c.1031G > A, p.(Gly344Asp); c.2296G > A, p.Ala766Thr)	c.1454_1462del GGCGGGAGA, p.(Arg485_Ile488del)	Known/known	Pathogenic/pathogenic
48	1	F	AD	26	cHSP	KIF5A	NM_004984.4	c.757A > C, p.(Lys253Gln)		Novel	Likely pathogenic
49	1	M	AR	20	pHSP	SPG7	NM_003119.4	c.233 T > A, p.(Leu78*)	c.233 T > A, p.(Leu78*)	Known/known	Pathogenic/pathogenic
50	1	F	AR	30	pHSP	SPG7	NM_003119.4	c.1529C > T, p.(Ala510Val)	c.228 T > C, p.(Ile743Thr)	Known/known	Pathogenic/pathogenic
51	1	M	AD	30	pHSP	SPAST	NM_014946.4	c.1390G > T, p.(Glu464*)		Known	Pathogenic
52	1	M	AD	38	cHSP	ALDH18A1	Nm_002860.4	c.755G > A, p.(Arg252Gln)		Known	Pathogenic
53	1	F	AD	42	pHSP	SPAST	NM_014946.4	c.1038_1039del, p.(Leu347Thrfs*14)		Known	Pathogenic
54	1	M	AR	54	cHSP	ATP13A2	NM_022089.4	c.3149_3152dup, p.(Ser1052Leufs*63)	c.1205C > T, p.(Thr402Met)	Novel/Known	Pathogenic/likely pathogenic
55	4	F	AD	20	pHSP	SPAST	NM_014946.4	Deletion exon 8		Known	Pathogenic

One representative patient from each family is shown. The second column indicates the number of examined patients per family. The table also includes the mode of inheritance, HSP form and age at onset. Variants are listed with gene name, transcript reference (NM number), and nucleotide as well as protein-level changes. For autosomal recessive forms, both alleles are shown. For these cases, information on whether each variant has been previously reported (e.g. *known/novel*) and the respective pathogenicity assessment (e.g. *pathogenic/likely pathogenic*) are provided for both alleles, separated by a slash

*pHSP* pure hereditary spastic paraplegia, *cHSP* complicated hereditary spastic paraplegia, *M* male, *f* female, *n* number, *AOA* age at onset, *AD* autosomal dominant, *AR* autosomal recessive, *X-L* X-linked, *Mito* mitochondrial

### Clinical characteristics

#### Baseline

Detailed clinical characteristics of 103 patients at the baseline visit were available. The most frequent complicating signs were sensory impairment (38.2%), ataxia (29.4%), and dysarthria (26.7%). Although not a complicating sign per the definition, 38.1% of the subjects had bladder disturbances. The frequency of urinary symptoms did not differ between the sexes or between the HSP forms.

**Fig. 2:**
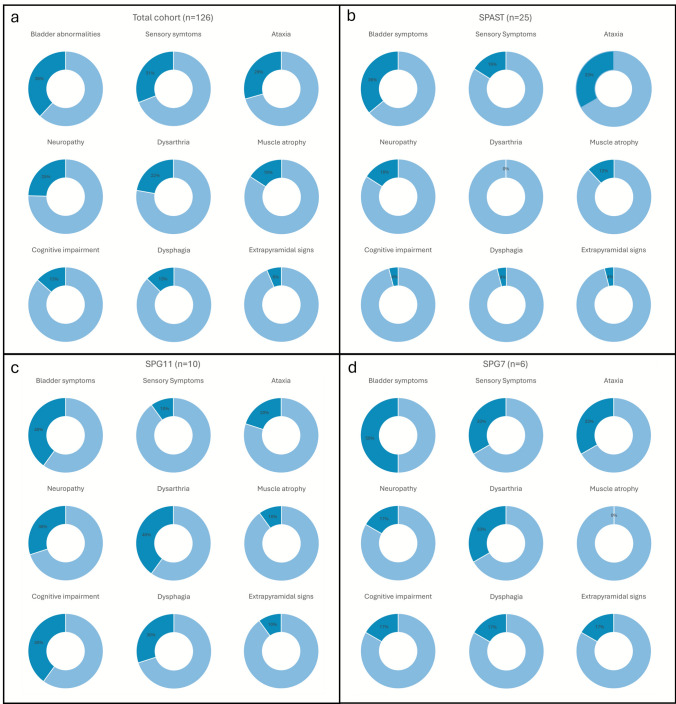
**Accompanying signs and symptoms.** Additional signs and symptoms present in the total cohort (**a**) as well as in the three most frequent genotypes, SPAST (**b**), SPG11 (**c**), and SPG7 (**d**), shown in decreasing frequency. Dark blue indicates the presence of the symptom; light blue indicates its absence. The percentage of patients presenting with each sign or symptom is indicated

For a full list of complicating signs and symptoms of the total cohort as well as those of the most frequent genotypes (*SPAST, SPG11* and *SPG7*), see Fig. 2.

The SPRS and 4SMD scores were available for 103 subjects. Disease severity, as defined by the mean SPRS score, of the total cohort was 18.2 (SD ± 10.5). There was no difference in SPRS between the forms or sexes. The 4SMD scale, defining mobility, showed that 68 (54.0%) patients could walk without aid (15 [11.9%] with mild symptoms, 53 [42.1%] without aid but unable to run), while 36 (28.6%) required walking aids and 22 (17.5%) were wheelchair bound. The median 4SMD score for pHSP was significantly lower than that for cHSP (2.0 [IQR 1] vs. 3.0 [IQR 1]; p = 0.001), reflecting a more severe disease in the latter. SPRS and 4SMD scores correlated significantly (Spearman-Rho 0.80, p < 0.001). There was no difference between sexes (p = 0.716) in terms of mobility.

#### Longitudinal data

**Fig. 3:**
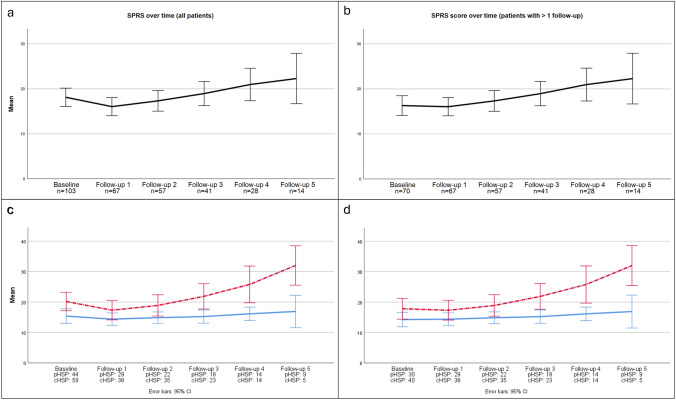
**SPRS-scores over time.** Progression of the SPRS score from baseline to follow-up 5. The number of available visits is indicated below each time point. In panels (**c**) and (**d**), numbers are shown separately for pHSP (blue and full line) and cHSP (red and dotted line). Panels (**a**) and (**c**) depict the total cohort, while panels (**b**) and (**d**) include only patients who attended more than one follow-up visit. Error bars represent 95% confidence intervals. The results shown are based on descriptive statistics rather than estimates from the generalized linear mixed model (GLMM). *pHSP* pure hereditary spastic paraplegia, *cHSP* complicated hereditary spastic paraplegia

Of the 103 patients with a baseline visit, 70 (55.6%) returned for at least one follow-up (FU). On average, the patients attended 2.3 (± 1.9) FU visits. FU5 was available in 14 patients who all stemmed from different families. Genetic testing was performed in all 14 patients, revealing a pathogenic variant in 8 (*SPAST* in 3 patients and *ABCD1, ATL1, SPG7, KIAA0196 and KIF5A* in 1 patient each), a VUS in 1 (*SPG7*), and no causative variant in the remaining 5 cases. Notably, patients who did not return for follow-up had higher disease severity at baseline, as measured by SPRS (22.0 ± 11.9 vs. 16.3 ± 9.2*; p* = 0.02) and reduced mobility according to 4SMD scores (3 [IQR 2] vs. 2 [IQR 1]; *p* = 0.042). This likely accounts for the seemingly paradoxical decrease in mean SPRS from 18.1 ± 10.4 at baseline to 16.0 ± 8.3 at follow-up visit 1 (Fig. 3a), a finding that is expected given that patients with more severe disease and mobility limitations are less likely to attend in-person visits. For mean SPRS scores at different visits see Figs. 3a, b, c and d.

Mean SPRS scores over time are shown in Fig. 3. Specifically, mean SPRS score change from baseline to year 1 was 0.8 ± 1.2 and from baseline to year 2 it was 1.8 ± 1.9. SPRS score changes during FU visits, overall and between forms, were assessed using a GLMM analysis. The results revealed a significant main effect of time (p < 0.001), indicating that SPRS scores changed significantly between FU visits. The estimated SPRS progression (i.e., percentage increase) per year for the total cohort was 5.1%, which translates to an increase of 0.9 points at a SPRS score of 18.2 (mean SPRS score at baseline). Furthermore, there was a significant interaction effect between the HSP form and FU visits (p < 0.001), suggesting that HSP forms followed different trajectories over time (Figs. 3c and d). Yearly progression for pHSP was 3.9%, corresponding to a yearly increase of 0.6 points at a baseline SPRS of 15.4 points, while it was 1.3 points (6.3%) at a baseline score of 20.2 points for cHSP. Disease duration was positively associated with SPRS progression (p < 0.001), whereas age at onset showed no significant effect (p = 0.673). To exclude a bias from missed visits, we repeated the GLMM analysis using the same parameters for the patients who completed a baseline to FU4 visits (complete-case-approach; n = 28). This analysis showed similar results with a comparable progression rate (5.1% per year in the total cohort vs. 5.0% in the complete-case-approach, both resulting in an increase of 0.9 points when calculated for the baseline SPRS), indicating robustness and representativeness of our results. As a final step, we also calculated the model for all SPG4 patients (n = 25), which again showed a significant increase in the SPRS score of 5.1%, corresponding to an average annual increase of 0.8 points from a baseline mean of 15.5 points.

### Real-world insights on patient management

Patient management data were evaluated in 126 patients with HSP. Overall, 32 patients (25.4%) were lost to follow-up, and 1 patient died.

#### Genetic testing

A diagnostic work-up in genetics was performed in 118 (93.7%) patients. Genetic testing was not performed in the remaining 8 patients (6.3%) because of loss to FU. Overall, 42 (33.3%) patients underwent single-gene testing, 53 (42.1%) underwent panel diagnostics, and 11 (8.7%) whole exome sequencing. Twelve patients (9.6%) were tested with multiple different tests. In comparison, single-gene testing was more predominant in pHSP (n = 28 [49.1%] vs. 14 [20.3%]; p < 0.001), while cHSP received panel diagnostics, exome sequencing, and multiple testing significantly more often (n = 52 [75.4%] vs. 24 [42.2%]; p < 0.001).

#### Education and employment

Education was primarily distributed across ISCED levels 2 to 5–9, with 68 of participants (54.5%) having completed upper secondary education (ISCED 3). Lower secondary education (ISCED 2) was reported by 17 participants (13.5%), while 11 participants (8.7%) had attained post-secondary non-tertiary education (ISCED 4). A total of 12 participants (9.5%) fell into the "academics" category (ISCED 5–9), reflecting higher education levels. Early education levels were rare, with 2 participants each in ISCED 0 and ISCED 1 (1.6% each), and educational status was unknown for 14 participants (11.1%). Educational level did not significantly differ between sexes (p = 0.559) nor forms (p = 0.056).

Employment status varied, with 36 patients (28.6%) being unable to work at the time of the last visit. The rate was significantly higher in the cHSP group than in the pHSP group (n = 27 [39.1%] vs. 9 [15.8%]; p = 0.003). No difference was observed between the sexes.

See Table 3 for detailed information on genetic testing as well as on education and employment of the cohort.

**Table 3:** Overview of real-world data (diagnostic, educational and employment) of the total cohort as well as stratified by HSP form

	Total	pHSP	cHSP
n (%)	126 (100)	57 (45.2)	69 (54.8)
Follow-up status, n (%)			
Alive	93 (73,8)	42 (73,7)	51 (73,9)
Deceased	1 (0,8)	1 (1,8)	0 (0)
Lost to follow-up	32 (25,4)	14 (24,6)	18 (26,1)
Genetic diagnostics, n (%)			
No testing	8 (6,3)	5 (8,8)	3 (4,3)
Single gene	42 (33,3)	28 (49,1)	14 (20,3)
Panel	53 (42,1)	20 (35,1)	33 (47,8)
Exome	11 (8,7)	2 (3,5)	9 (13)
Single and panel	6 (4,8)	1 (1,8)	5 (7,2)
Single and exome	1 (0,8)	0 (0)	1 (1,4)
Panel and exome	2 (1,6)	1 (1,8)	1 (1,4)
All	3 (2,4)	0 (0)	3 (4,3)
Employment status, n (%)			
ISCED 0	2 (1,6)	1 (1,8)	1 (1,4)
ISCED 1	2 (1,6)	0 (0)	2 (2,9)
ISCED 2	17 (13,5)	3 (5,3)	14 (20,3)
ISCED 3	68 (54)	33 (57,9)	35 (50,7)
ISCED 4	11 (8,7)	4 (7)	7 (10,1)
ISCED 5–8	12 (9,5)	7 (12,3)	5 (7,2)
Unknown	14 (11,1)	9 (15,8)	5 (7,2)
Employment status, n (%)			
Unemployed	36 (28,6)	9 (15,8)	27 (39,1)
Employed	52 (41,3)	26 (45,6)	26 (37,7)
Retired	23 (18,3)	14 (24,6)	9 (13)
Child	2 (1,6)	0 (0)	2 (2,9)
Unknown	13 (10,3)	8 (14)	5 (7,2)

Data are presented as number (percentage) within each group

*pHSP* pure hereditary spastic paraplegia, *cHSP* complicated hereditary spastic paraplegia, *ISCED* international standard classification of education

#### Neuroimaging, neurorehabilitation and management

**Fig. 4:**
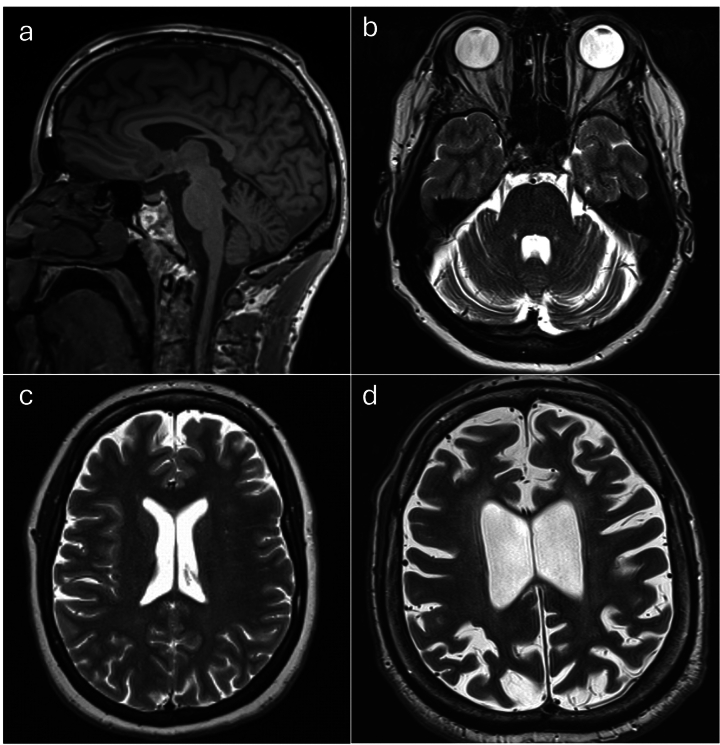
**MRI findings.** This figure highlights some illustrative MRI findings of the cohort. **a** demonstrates the novel finding of a thin corpus callosum in a subject with a *WASHC5* mutation. **b** depicts cerebellar atrophy in a subject with *SPG7*. **c** and d highlight cerebral atrophy in *SPG10* and *SPG11*, respectively

MRI scans were available for 76% of patients. Approximately one-third of patients (n = 41, 32.5%) underwent cerebral MRI only, 53 patients (42.1%) received both cerebral and spinal MRI, and two patients (1.6%) underwent spinal MRI only. Cerebral and cerebellar atrophy were each observed in approximately 40%, and a thin corpus callosum (TCC) in 13% of cases. One of the subjects with TCC carried a mutation in the SPG8 gene (see Fig. 4 for illustrative MRI images).

Symptomatic treatment was prescribed in 70% of patients, most commonly anti-spastic medication, and 84% received neurorehabilitation. Multimodal rehabilitation was more frequent in cHSP than in pHSP (p = 0.032). Detailed MRI and therapy data are provided in the supplementary files.

## Discussion

### Demographics and real-world insights on management of HSP in Austria

This study provides comprehensive real-world data on a large Austrian HSP cohort from the Center for Rare Movement Disorders Innsbruck (CRMDI), which serves as the Austrian reference center for rare movement disorders and is a part of the European Reference Network for Rare Neurological Diseases. This constitutes the most comprehensive natural history data of an Austrian HSP cohort to date.

Multiple HSP cohort studies have mainly focused on epidemiological or genetic assignment rather than real-world management data [8, 10, 11]. There are two large cohorts from Germany and France based on comprehensive genetic and clinical assessments [3, 4]. Our HSP cohort was comparable regarding most of the demographic variables such as age at onset or disease duration. Moreover, the distributions of pure HSP (pHSP) and complicated HSP (cHSP) were similar. The large European HSP cohorts exhibited a male predominance [3, 4], which was even more pronounced in the Austrian cohort.

Genetic testing was performed in 118 patients with HSP in our study. Single-gene testing was performed more frequently in pHSP. This is most likely due to the fact that pHSP forms, in accordance with the literature, are mostly inherited in an AD manner [12]. In simplex or unsolved cases, NGS gene panels and WES were performed more frequently. Overall, this resulted in a genetic confirmation rate of 54% in HSP patients in our cohort, which was significantly higher than that in French (31%) (p < 0.001) but not the German (49%) cohorts [3, 4]. When stratified by inheritance pattern, genetic confirmation was achieved in 34 of 46 familial HSP index cases (73.9%), compared with 15 of 56 sporadic index cases (26.8%). One reason for the higher genetic confirmation rate of our cohort is the availability of genetic testing using new sequencing methods, such as exome sequencing. In addition to differences in the rate of genetic confirmation, the modes of inheritance also differed between cohorts. Simplex cases were most prevalent in the French [3], less common in the Austrian and least frequent in the German cohort [4]. Notably, our Austrian cohort demonstrated the highest rate of recessive HSP genes (17 families with compound heterozygous mutations and 11 families with homozygous mutations, thereof 7 consanguine), accounting for almost one-third of the cases, compared to only 10–12% of recessive inheritance in the other cohorts [3, 4]. The rate of consanguinity in the French cohort was almost twice as high as in our cohort [3]. In these cohorts, *SPAST* was the most common genotype, followed by *SPG7*, and *SPG11*. While *SPAST* was also the most frequent genotype in our cohort, we found more cases with SPG11 than with SPG7. This was in contrast to the findings in several cohorts [3, 4, 13, 14] and may depict a genotype distribution specific to Austria. Interestingly, two unrelated SPG21 families were part of our cohort. SPG21 (ACP33 gene) was originally described in Amish and is rarely observed outside of this population. Our pedigrees have been previously published in detail [15]. Also noteworthy are the four subjects with mutations in the ABCD1 gene. Three were male, and none were related. Very long chain fatty acids were elevated in all patients, confirming pathogenicity of the mutations. All presented with an adrenomyeloneuropathy phenotype, clinically resembling HSP [16].

MRI scans were available in 76% of patients. Overall, approximately one third of patients underwent cerebral MRI only, while just over 40% received both cerebral and spinal MRI. In contrast, spinal MRI alone was performed in only a small minority of cases (1.6%). Abnormal findings were more frequent than previously reported, with 61.4% of cerebral and 11.4% of spinal MRI showing pathology, compared to 28% in a Canadian cohort and 34.4% in a French cohort [3, 17]. Cerebral and cerebellar atrophy were each observed in over 40% of patients, and a thin corpus callosum (TCC) in 13%, including one patient with a *SPG8/WASHC5* mutation, which is a novel finding for this mutation [3] (see Fig. 4a). The high rate of findings underscores the need for standardized MRI protocols.

Over 70% of patients received pharmacological treatment, most commonly anti-spastic medication, though discontinuation due to limited efficacy was frequent. Botulinum neurotoxin was applied in only 4% of patients. Overall, just over half of the patients were on antispastic medication despite low threshold access in our healthcare system, highlighting the limited effectiveness of current therapies. Urinary symptoms were reported by 38%, yet only 14% received treatment, with no female predominance observed, unlike prior reports [18]. Given the well-established impact of urinary symptoms on the quality of life of patients [18–20], this highlights the opportunity for improvement in the screening and assessment of urinary symptoms in HSP patients.

Apart from pharmacological treatment, rehabilitation is another key component of HSP management. The beneficial effects of several rehabilitative measures, including modern approaches such as robotics, have been demonstrated [21, 22]. Our cohort had a high rate of patients participating in neurorehabilitative therapy, with only 15.9% not receiving any form of therapy. Patients with cHSP received regular therapy significantly more often than patients with pHSP.

Detailed MRI and therapy data are provided in the supplementary material.

### Clinical characteristics and progression

Functional impairment in our cohort was assessed using the 4SMD. Since other HSP scales do not include a functional outcome tool, we introduced the 4SMD [8, 23–25]. In our cohort, more than 50% of patients with HSP did not use assistive devices, while 17.5% of patients were wheelchair-bound. In the French cohort, a comparable scale called the SPATAX functional score was used. The SPATAX score ranges from 0 (no functional impairment) to 7 (confined to bed) [3]. The median score in this cohort was 3, corresponding to moderate impairment in which subjects were unable to run and walking without an aid was limited. While not directly comparable, this corresponds well to our median 4SMD score of 2, which also equals subjects being unable to run but mobile without walking aids.

The SPRS is a comprehensive, validated HSP rating scale that allows for a detailed depiction of disease severity in HSP. This scale has been used in several international cohort studies and interventional trials. The mean scores in these cohorts ranged from 17.4 to 19.9 points [4, 23, 26, 27]. In our cohort, disease severity was representative, with a mean score of 18.2 points on the SPRS. Additionally, the 4SMD scores significantly correlated with the SPRS scores in our patients. Therefore, our cohort reflects previously published HSP cohorts regarding disease severity. In addition to cross-sectional SPRS data, we first presented longitudinal follow-up measures with SPRS in an adult HSP cohort [28]. In the present study, we used a GLMM with SPRS as a dependent variable in our cohort, showing a significant increase in SPRS scores during FU visits, resulting in an average annual progression rate of 0.9 points in SPRS. Additionally, there were significant differences between the two HSP forms, with faster disease progression in cHSP (1.3 points vs. 0.6 points). To exclude potential bias from missed visits at later FU, we conducted a complete-case analysis, which yielded progression rates identical to those of the full-cohort model, supporting the robustness of our findings. While disease duration showed a significant effect on SPRS increase over the course of the study, as it would be expected, no such effect was observed for age at onset. This finding contrasts with previously published cross-sectional data, in which a later disease onset was associated with faster disease progression [4]. The reason for this discrepancy remains unclear and should be explored in future studies.

To the best of our knowledge, this is the first natural history cohort study prospectively assessing the longitudinal capabilities of the SPRS, thereby addressing a significant knowledge gap in the current understanding of outcome measures in HSP. Our findings show that the SPRS reliably depicts disease progression over time. However, for a comprehensive assessment of disease burden, patient-reported outcome measures (PROM) remain essential. As the SPRS does not incorporate PROM, these should be evaluated in parallel. In a previous study we have shown, that some generic PROM have limited capabilities in capturing disease-specific aspects of HSP [23]. Since then, HSP-specific PROM [29, 30] have been developed. However, their performance over longer observational periods have not yet been systematically studied. This highlights the need for further studies to explore the combined use of CROM and PROM in long-term evaluations of HSP.

In addition to the detailed evaluation of the SPRS, our cohort also contributes to a broader understanding of HSP characteristics across different populations. The distribution of pHSP and cHSP rates was comparable to those of French and German HSP cohorts, while the frequency of complicating signs and symptoms varied [3, 4]. Some additional symptoms in our series, such as ataxia (29%), were comparable with the German (29%) and French cohorts (34%). However, dysarthria (25%) was the more common in our cohort, whereas the rate of dysarthria was lower in two comparable European HSP cohorts. Dysphagia was almost twice as common in our cohort. There is no evident reason for this disparity in several HSP cohorts, and it may be subject to future assessments and evaluations. In contrast, extrapyramidal signs were almost four times more frequent in the French cohort than in our cohort and the German HSP cohort [3, 4]. While these larger studies have helped characterize the clinical spectrum of HSP, they lacked prospective longitudinal data using standardized clinical outcome measures such as the SPRS. Our study addresses this gap and further highlights the phenotypic heterogeneity of HSPs, as well as the necessity of standardized assessment methods for additional symptoms with a high functional impact on patients.

In summary, we present the results of a large Austrian HSP cohort with an in-depth clinical and genetic characterization and prospectively assessed for a period of up to five years. This is the first prospective evaluation of the widely used SPRS in an HSP cohort study. We were able to show that the SPRS score increased significantly during the follow-up period. While this is an important first step for the planning of possible upcoming future trials, it also highlights the importance of standardizing assessments of genetics, clinical characteristics, and biomarkers of large HSP cohorts in order to further validate our results and to be prepared for possible upcoming interventional trials.

## Supplementary Information

Below is the link to the electronic supplementary material.

Supplementary file1 (DOCX 35 KB)

## Data Availability

Data is available upon request.
