# Supplementary material for: Natural history in hereditary spastic paraplegias: real-world data from an Austrian cohort
Source: J Neurol. 2026 Jan 26;273(2):97. doi: 10.1007/s00415-025-13606-y (PMC12835074; doi:10.1007/s00415-025-13606-y)
Supplement: Supplementary file 1 — Supplementary file1 (DOCX 35 KB) [file 415_2025_13606_MOESM1_ESM.docx]

Supplementary material

# Real-World Insights on Patient Management

This section provides detailed information on magnetic resonance imaging (MRI) findings and therapeutic management in the study cohort.

## Magnetic resonance imaging (MRI)

MRI scans (in-house) were performed in 96 (76.2%) patients, with approximately one-third (n=41, 32.5%) receiving cerebral MRI (cMRI), and 53 (42.1%) receiving both spinal (sMRI) and cerebral MRI. Two patients (1.6%) underwent only sMRI. The number of patients without MRI was higher in pHSP (n=22 [38.6%] vs. 8 [11.6%]; p=0.002), mainly due to fewer cMRI scans in family members from large AD families (n=11 [19.3%] vs. n=30 [43.3%]; p=0.004). While several MRI pathologies are widely recognized as HSP-associated and multiple studies have focused on specific MRI findings in distinct genotypes (1, 2), only limited data on the frequency of MRI findings in a clinical routine setting are available. In our cohort, of the 94 patients with cMRI, 59 (61.4%) and of the 55 patients with sMRI, 11 (11.4%) had abnormal results. Both cerebral and cerebellar atrophy were present in more than 40% of patients. TCC was present in 12.8% of cases. While three subjects had SPG11/SPATACSIN and two had *SPG21/ACP33*, both associated with TCC (1, 3, 4) one patient harboured a mutation in *SPG8/WASHC5*, which was a novel finding in this mutation (5). The remaining six patients with TCC were genetically unsolved (see Figure 5 main manuscript for illustrative images). The high rate of MRI findings highlights the need to harmonize future MRI assessments, e.g., using MR volumetry and tractography protocols.

Detailed MRI data are presented in supplementary table 1.

## Therapy and management

Over 70% of patients received pharmacological treatment, most commonly anti-spastic medication. Many patients without antispastic therapy had discontinued previous treatment attempts due to limited or no benefit. Botulinum neurotoxin was applied to five patients (4%) in a limited therapeutic window. Overall, just over half of patients received antispastic medication despite easy access, highlighting limited efficacy of current therapies and the need for treatments targeting underlying pathophysiology. Furthermore, 38.1% of patients reported urinary symptoms, but only 14% received treatment. Urinary complaints showed no female predominance, in contrast to earlier studies (6). Given their impact on quality of life (6-8), better screening and assessment are warranted.

Rehabilitation remains a key component of HSP management. Beneficial effects of several rehabilitative measures, including robotics, have been demonstrated (9, 10). Our cohort had a high participation rate in neurorehabilitative therapy, with only 15.9% receiving none. Patients with cHSP received regular therapy more often than those with pHSP (cHSP n=63 [91.3%] vs. pHSP n=43 [75.4%]; p=0.032).

Detailed therapy data are provided in supplementary table 2.

**Supplementary Table 1: Real-world data on MRI**

|  | | Total | pHSP | cHSP |
| --- | --- | --- | --- | --- |
| n (%) |  | 126 (100) | 57 (45.2) | 69 (54.8) |
| Magnetic resonance imaging, n (%) | |  |  |  |
| Not done | | 30 (23,8) | 22 (38,6) | 8 (11,6) |
| Cerebral | | 41 (32,5) | 11 (19,3) | 30 (43,5) |
| Spinal | | 2 (1,6) | 1 (1,8) | 1 (1,4) |
| Both | | 53 (42,1) | 23 (40,4) | 30 (43,5) |
| MRI findings, n (%) | |  |  |  |
| Normal | | 34 (35,4) | 15 (42,9) | 19 (31,1) |
| Abnormal cerebral | | 51 (53,1) | 15 (42,9) | 36 (59) |
| Abnormal spinal | | 3 (3,1) | 2 (5,7) | 1 (1,6) |
| Both abnormal | | 8 (8,3) | 3 (8,6) | 5 (8,2) |

**Supplementary Table 1:** Overview of MRI data of the total cohort as well as stratified by HSP form. Data are presented as number (percentage) within each group.

pHSP = pure hereditary spastic paraplegia; cHSP = complicated hereditary spastic paraplegia; MRI = magnetic resonance imaging.

**Supplementary Table 2: Real-world data on therapy**

|  | Total | pHSP | cHSP |
| --- | --- | --- | --- |
| Medication, n (%) |  |  |  |
| No medication | 38 (30.2) | 16 (28.1) | 22 (31.9) |
| Antispastic drugs | 69 (54.8) | 33 (57.9) | 36 (52.2) |
| Antidepressants | 32 (25.4) | 10 (17.5) | 22 (31.9) |
| Urinary | 18 (14.3) | 11 (19.3) | 11 (10.1) |
| BoNT | 5 (4.0) | 2 (3.5) | 3 (4.3) |
| Number of medications, n (%) |  |  |  |
| No medication | 38 (30.2) | 16 (28.1) | 22 (31.9) |
| One category | 57 (45.2) | 28 (49.1) | 29 (42.0) |
| Two categories | 26 (20.6) | 11 (19.3) | 15 (21.7) |
| Three categories | 5 (4.0) | 2 (3.5) | 3 (4.3) |
| Rehabilitation/therapy |  |  |  |
| No therapy | 20 (15.9) | 14 (24.6) | 6 (8.7) |
| Outpatient therapy | 42 (33.3) | 17 (29.8) | 25 (36.2) |
| Inpatient therapy | 2 (1.6) | 2 (3.5) | 0 (0.0) |
| Both | 62 (49.2) | 24 (42.1) | 38 (55.1) |

**Supplementary Table 2**: Overview of pharmacological and rehabilitation management. In the first section, all pharmacological treatments received by the patients are listed; as several patients received multiple therapies and were counted separately for each, the total exceeds 100%. The number of medications as well as Overview of treatment strategies of the total cohort as well as the rehabilitation data are presented as number (percentage) within each group.

pHSP = pure hereditary spastic paraplegia; cHSP = complicated hereditary spastic paraplegia; BoNT = botulinum neurotoxin.

1. Blackstone C. Hereditary spastic paraplegia. Handb Clin Neurol. 2018;148:633-52.

2. Servelhere KR, Rezende TJR, de Lima FD, de Brito MR, de França Nunes RF, Casseb RF, et al. Brain Damage and Gene Expression Across Hereditary Spastic Paraplegia Subtypes. Movement Disorders. 2021;36(7):1644-53.

3. Amprosi M, Indelicato E, Nachbauer W, Hussl A, Stendel C, Eigentler A, et al. Mast Syndrome Outside the Amish Community: SPG21 in Europe. Front Neurol. 2021;12:799953.

4. Rattay TW, Schöls L, Zeltner L, Rohrschneider WK, Ernemann U, Lindig T. "Ears of the lynx" sign and thin corpus callosum on MRI in heterozygous SPG11 mutation carriers. J Neurol. 269. Germany2022. p. 6148-51.

5. Méreaux JL, Banneau G, Papin M, Coarelli G, Valter R, Raymond L, et al. Clinical and genetic spectra of 1550 index patients with hereditary spastic paraplegia. Brain. 2022.

6. Schneider SA, Beckinger VE, Möller B, Knüpfer S, Hamann M, Deuschl G. Urinary symptoms, quality of life, and patient satisfaction in genetic and sporadic hereditary spastic paraplegia. Journal of neurology. 2019;266(1):207-11.

7. Braschinsky M, Zopp I, Kals M, Haldre S, Gross-Paju K. Bladder dysfunction in hereditary spastic paraplegia: what to expect? Journal of Neurology, Neurosurgery & Psychiatry. 2010;81(3):263-6.

8. Lallemant-Dudek P, Guillaud-Bataille M, Hentzen C, Joussain C, Pichon B, Robain G, et al. Hereditary spastic paraplegias: When to expect bladder dysfunction a genetic and urodynamic study. European Journal of Neurology. 2025;32(1):e70003.

9. Bellofatto M, De Michele G, Iovino A, Filla A, Santorelli FM. Management of Hereditary Spastic Paraplegia: A Systematic Review of the Literature. Front Neurol. 2019;10:3.

10. Bertolucci F, Di Martino S, Orsucci D, Ienco EC, Siciliano G, Rossi B, et al. Robotic gait training improves motor skills and quality of life in hereditary spastic paraplegia. NeuroRehabilitation. 2015;36(1):93-9.
